# Season-specific effects of α-tocopherol supplementation during bovine oocyte *in vitro* maturation on embryo yield and quality

**DOI:** 10.1590/1984-3143-AR2024-0136

**Published:** 2025-05-09

**Authors:** Bruno Barcelona, Zully Ramos, Carolina Viñoles, Nélida Rodríguez-Osorio, Francisco Báez

**Affiliations:** 1 Instituto Superior de la Carne, Centro Universitario Regional – CENUR Noreste, Universidad de la República – UDELAR, Tacuarembó, Uruguay; 2 Centro de Salud Reproductiva de Rumiantes en Sistemas Agroforestales – CENUR Noreste, Universidad de la República – UDELAR, Cerro Largo, Uruguay; 3 Unidad de Genómica y Bioinformática, Departamento de Ciencias Biológicas – CENUR Litoral Norte, Universidad de la República – UDELAR, Salto, Uruguay

**Keywords:** temperature-humidity index, blastocysts, ROS, apoptotic, gene expression

## Abstract

Elevated temperature-humidity index (THI) levels, common in subtropical summers, can impair bovine oocyte development by increasing reactive oxygen species (ROS) accumulation, leading to oxidative stress and reduced developmental competence. Alpha-tocopherol, a potent antioxidant, has the potential to mitigate these effects by scavenging ROS. However, its seasonal efficacy during bovine oocyte *in vitro* maturation (IVM) remains underexplored. This study evaluated the impact of 100 µM α-tocopherol supplementation during IVM on oocytes collected in spring (THI: 68.7±3) and summer (THI: 73±3) in Northern Uruguay. Oocytes underwent IVM, fertilization, and embryos were cultured *in vitro* until day 9 post-fertilization. Blastocysts were assessed for ROS levels, apoptosis, and the abundance of transcripts linked to development and oxidative stress. Results showed a season-specific response to α-tocopherol supplementation. While no significant effects were observed in spring, summer oocytes exhibited increased maturation, cleavage, and blastocyst rates, along with improved blastocyst quality characterized by reduced apoptosis and lower *BAX* transcript levels. These findings indicate that α-tocopherol supplementation during IVM enhances oocyte developmental competence under heat stress conditions, supporting its potential as a strategy to mitigate oxidative damage and improve bovine embryo production during summer.

## Introduction

The livestock industry is facing difficulties due to global warming (Lovarelli et al., 2024). Climate change has become evident through the increased frequency of heat waves in late spring and summer (Hassan et al., 2024). This is particularly interesting given that the breeding season of subtropical pastoral system beef cows occurs in late spring and earlier exposure to heat waves and higher Temperature-humidity Index (THI) may generate heat stress. When the THI rises above 72.8 cows experiment a linear increase in vaginal temperature, which could lead to lower pregnancy rates (Fedrigo et al., 2021).

In a seasonal study, we reported compromised oocyte and embryo quality during summer, evidenced by increase DNA fragmentation levels in cumulus-oocyte complexes (COCs) and reduced blastocyst rates, compared to winter (Báez et al., 2022). This lower developmental capacity for summer oocytes could be associated with hyperthermia-induced increased production of reactive oxygen species (ROS). Oocyte hyperthermia during summer can be considered one of the main causes of increased embryonic ROS content and oxidative stress (Deluao et al., 2022), which cause lipid peroxidation, DNA fragmentation, and mitochondrial dysfunction (Guérin et al., 2001), leading to embryonic delay, arrest, or death.

Antioxidants, such as α-tocopherol (vitamin E), are known to counteract oxidative damage by scavenging peroxyl radicals and preventing lipid peroxidation. Its incorporation into culture media has been associated with a higher *in vitro* blastocyst rates and quality in several species (Arias-Álvarez et al., 2018; Maddahi et al., 2024; Tripathi et al., 2023). Recently, we reported that the addition of 100 μM α-tocopherol during *in vitro* maturation (IVM) improves the developmental competence of bovine oocytes collected during summer, leading to improved embryo yield, along with increased expression of *SOD2* in blastocysts (Báez et al., 2021). However, it remains unclear whether α-tocopherol´s beneficial effects on the expression of blastocyst genes, such as those in involved stress response (*HSPA1A and CAT*), apoptotic (*BAX*), maternal recognition of pregnancy (*IFNT2*), membrane transport (*AQP3*), and pluripotency (*OCT4*), also extend to oocytes collected in cooler seasons, such as spring. We hypothesize that supplementing IVM media with 100 μM α-tocopherol improves the developmental competence of bovine oocytes not only in summer but also in spring, by reducing oxidative stress, and increasing the expression of genes associated with embryo quality. Therefore, this study aimed to evaluate the effect of α-tocopherol supplementation during IVM on the developmental competence of bovine oocytes collected during both the austral spring and summer seasons.

## Methods

This study did not require ethical committee approval since it did not involve direct experimentation on live animals. Bovine COCs were aspirated from the ovaries of Hereford, Angus, and Hereford x Angus crossbred cows, which are non-lactating and maintained on a high plane of nutrition to achieve a body condition above 5 (scale 1 to 8; Vizcarra and Ibañez, 1986). Sampling was conducted from November 10 to December 15, 2023, representing the austral spring, and from January 16 to February 22, 2024, representing the austral summer. THI was calculated using the formula described by Thom (1959):


$$THI=\left(0.8x\;T+\left(RH/100\right)\left(T-14.4\right)+46.4\right)$$ (1)

T: air temperature in degrees Celsius, RH: relative humidity (%).

A total of 1,466 immature COCs were collected and assigned to two groups per season: maturation media TCM-199 supplemented with 0.05% (v/v) ethanol (control group) or 100 μM α-tocopherol in 0.05% (v/v) ethanol (treatment group) for 24 h. After IVM, 59-68 COCs per group were denuded, fixed and stained to determine maturation rate. Then, 1,218 matured COCs were fertilized *in vitro*. From those, 47-76 presumptive zygotes per group were denuded, fixed, and stained to determine fertilization rate. The remaining presumptive zygotes, 232-295 per group, were cultured in synthetic oviduct fluid (SOF) media. Embryo development rates were recorded at days 3 and 9 post-insemination. Expanded blastocysts from all treatments were used for ROS assessment (9-10 per group), apoptotic index with *Terminal deoxynucleotidyl transferase dUTP Nick-end labeling* (TUNEL) staining (7 per group), and gene expression analysis (3 pools of up to 2 blastocysts per group). Three replicates were performed per season. The model included season, treatment, and their interaction as fixed effects.

### *In vitro* maturation

COCs were aspirated from 2-8 mm follicles and washed twice in HEPES medium (HM, TCM199 Sigma-M7528) with 5 mg/mL BSA. Groups of 50 COCs with compact layers of cumulus cells and homogeneous cytoplasm were cultured in four-well dishes with 500 μL of maturation medium TCM199 (Gibco-11150059) supplemented with 10% FCS, 1 μg/mL FSH (Bioniche-Folltropin-V^®^), 5 UI/mL of eCG (Biogón^®^), 0.2 mM sodium pyruvate, 50 µg/mL gentamicin, 100 μM α-tocopherol in 0.05% (v/v) ethanol, and covered with mineral oil for 24 h at 38.5ºC with 5% CO_2_, and air balanced with N_2_. A control group with the addition of 0.05% ethanol (vehicle) was included for both seasons for a total of four groups: 1) Spring control, 2) Spring α-100, 3) Summer control, and 4) Summer α-100.

### *In vitro* fertilization (IVF) and embryo development

For IVF, frozen straws from a fertile Angus bull were thawed at 38ºC and added to 1 mL of TALP (Tyrode’s Albumin-Lactate-Pyruvate-solution) supplemented with 10 mM HEPES and incubated for 1 hour at 38.5ºC, 5% CO_2_. Then, 800 μL of supernatant was centrifuged for 8 min at 250 xg. The sperm pellet was resuspended in TL-IVF (TALP solution supplemented with 10 μg/mL heparin and 10 μM hypotaurine). Matured COCs were washed twice in drops of TL-IVF and co-incubated with 1x10^6^ sperm/mL in 500 µL of TL-IVF for 20 h at 38.5ºC with 5% CO_2_. After fertilization, presumptive zygotes were denuded of cumulus cells in HM and washed twice in SOF media containing nonessential and essential amino acids, 0.34 mM citrate, 2.7 mM myo-inositol, 6 mg/mL BSA and 2.5% FCS (Holm et al., 1999). Presumptive zygotes were cultured in ~25μL droplets of SOF (1 μL/embryo) at 38.5°C, 5% CO_2_, 5% O_2_, and 90% N_2_ for 9 days.

### Maturation and fertilization rate assessment

Matured COCs and presumptive zygotes were denuded and fixed in 2.5% paraformaldehyde (wt/vol) and stained with 10 μg/mL of bisbenzimide for 5 min, then mounted on slides with 5 μL of PBS and examined under a fluorescence microscope. Oocytes were classified as mature (metaphase II + polar body) and fertilized (two pronuclei) as described by Báez et al. (2021).

### ROS production

Expanded blastocysts were transferred into SOF medium with 10 μM 2´7´-dichrolorohydro-fluorescein diacetate (H_2_DCFDA, D6883 Sigma-Aldrich) at 5% CO_2_ for 30 min. Then, blastocysts were washed twice in PBS for 5 min and examined in a Nikon Eclipse 50i fluorescence microscope equipped with UV filters (460 nm). ROS levels were quantified using ImageJ software (Yoon et al., 2014).

### Apoptosis assessment

Blastocysts were fixed using 2.5% paraformaldehyde for 1 h and washed in PBS. DNA fragmentation was detected using an In Situ Cell Death Detection Kit (Roche Diagnostics Corp., Indianapolis, USA) according to the manufacturer’s protocol. Fixed blastocysts were permeabilized with 0.5% Triton X**-**100 for 45 min at room temperature and incubated in the dark with TUNEL reaction solution for 1 h at 37°C. Samples were washed and stained with 10 μg/mL bisbenzimide for 5 min and mounted onto a glass slide. Blastocysts were analyzed in a fluorescence microscope. The apoptotic index was calculated as a percentage of FITC positive cells (labeled green) within all detected Hoechst cells (Rodrigues et al., 2016).

### Gene expression analysis

Blastocysts from each group were washed and frozen in liquid nitrogen. Total RNA was extracted using reagents from the Single Cell RNA Purification Kit, (Norgen Biotek Corp. Canada) and quantified using a Nanodrop spectrophotometer. cDNA was synthesized from 15 ng of total RNA per sample using SuperScriptIII transcriptase (Thermo Fisher Scientifi, Waltham, MA, USA). Quantitative polymerase chain reaction (qPCR) was performed using a Mic-qPCR (Bio Molecular System, Australia) thermal cycler, SYBR Green master mix (Thermo Fisher Scientific, Waltham, MA, USA) and the primers presented in Table 1. Standard amplification conditions were 95°C for 5 min followed by 40 cycles of 95°C for 15 s, 58°C for 40 s, and 72°C for 20 s. Relative expression was calculated using the 2^-ΔΔCT^ method (Livak and Schmittgen, 2001) and normalized against the reference genes *ACTB* and *GAPDH*. Data were derived from three independent replicates for each gene.

**Table 1:** Oligonucleotide primers for RT-qPCR analysis.

**Gene symbol**	**Primer sequence (5´-3´)**	**Fragment size (bp)**	**GenBank Accession No.**
*AQP3*	F: ACCGATCTAGCCCCTCATCT	136	NM_001079794
	R: CCAACTCCACCGACAGAATC		
*HSP1A1*	F: CTTCAACATGAAGAGCGCCG	182	NM_203322.3
	R: TGATGGGGTTACACACCTGC		
*OCT4*	F: AGTGAGAGGCAACCTGAAGA	110	NM_174580.2
	R: ACACTCGGACCACGTCTTTC		
*IFNT2*	F: TCTGAGGACCACATGCTAGG	145	NM_001015511.3
	R: GATCCTTCTGGAGCTGGTTG		
*BAX*	F: TTTGCTTCAGGGTTTCATCCA	126	NM_173894.1
	R: CCGATGCGCTTCAGACACT		
*CAT*	F: GTTCGCTTCTCCACTGTT	454	NM_001035386.2
	R: GGCCATAGTCAGGATCTT		
*ACTB*	F: GACATCCGCAAGGACCTCTA	205	NM_173979
	R: ACATCTGCTGGAAGGTGGAC		
*GAPDH*	F: GATTGTCAGCAATGCCTCCT	94	NM_001034034.2
	R: GGTCATAAGTCCCTCCACGA		

Abbreviations: F: forward; R: reverse.

### Statistical analysis

Data were analyzed utilizing the General Linear Models (GLM) procedure in SAS (SAS Institute, Cary, NC, USA). The dependent variables included the percentage of matured, fertilized, and divided oocytes, percentage of blastocysts, number of blastomeres, ROS production, apoptotic index, and relative gene expression. Data were analyzed using a one-way ANOVA with Tukey’s test. Effects of season, treatment, and their interaction were analyzed by two-way ANOVA using the Tukey-Kramer multiple comparison test. For THIs, the effect of season was tested for significance. Differences between means were considered significant when *P* <0.05.

## Results

Mean THI was higher in the summer than in late spring (*P*<0.01). Maturation and fertilization did not differ in spring oocytes after α-tocopherol supplementation. However, in summer oocytes, significant differences (*P*<0.05) were observed between the control and α-100 groups, with the latter showing values similar to those of spring oocytes. Summer control oocytes also produced the lowest embryo rates (*P*<0.05), as shown in Table 2. Despite this, no differences in embryo cell numbers were detected across the groups.

**Table 2:** Maturation (metaphases II), fertilization, cleavage and blastocysts rates (percentage, % ± s.e.m) in bovine oocytes collected during spring and summer and *in vitro* matured without (control) or with 100 μM α-tocopherol (α-100). Temperature–humidity index (THI) was calculated during austral late spring (between 10^th^ of November to 15^th^ of December 2023) and summer (between 16^th^ of January to 22^nd^ of February 2024).

**Variable**	**Spring (THI: 68.7±3)**		**Summer (THI: 73±3)**		**Interaction**
	**Control**	**α-100**	**Control**	**α-100**	**Season x** **Antioxidant**
Metaphase II (%)	83±3%^a^	81.4±1%^a^	75±1%^b^	83.5±0.8%^a^	**
Fertilization rate (%)	74.5±2%^a,b^	76±2.5%^a^	68.5±1%^b^	77.6±2%^a^	NS
Cleavage rate (Day 3)	73.8±1%^a,b^	75.5±2%^a^	70.7±1%^b^	78±2.5%^a^	*
Blastocysts rate (Day 9)	27.2±1%^a^	28.8±1%^a^	19.8±4%^b^	27±5%^a^	NS

Different letters (a, b) within rows indicate significant differences between treatments (*P < 0.05*). NS= not significant; ^*^ *P*<0.05; ^**^ *P*<0.001.

**Figure 1:**
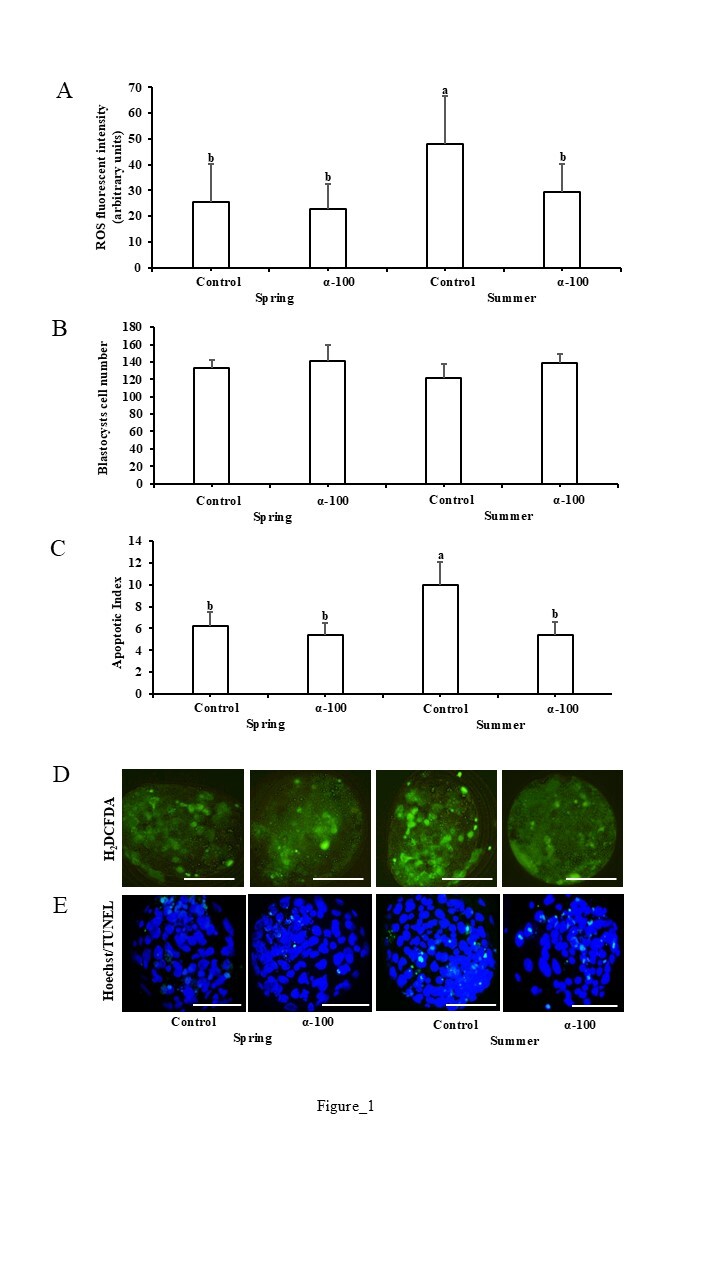
Effect of 100 µM of α-tocopherol supplementation during bovine oocyte *in vitro* maturation during spring and summer on (A) reactive oxygen species (ROS) production, (B) blastocysts cell number and (C) expanded blastocyst apoptotic index. (D) Fluorescence images of Day 9 expanded blastocysts treated with 2´7´-dichrolorohydro-fluorescein diacetate (H_2_DCFDA) for evaluated ROS production. (E) Merge images of Day 9 expanded blastocysts stained with Hoechst 33342 (blue nuclei) and TUNEL (green) for evaluated DNA fragmentation. The white bar represents the range of 100 μm. The absence of letters in each bar represents no significant differences. Different letters in each bar represent significant differences (*P < 0.05*).

The interaction between season and antioxidant significantly affected the apoptotic index (*P*<0.01) and *BAX* expression levels (*P*<0.03). As shown in Figure 1, the highest levels of ROS production (*P*<0.05) and apoptosis index (*P*<0.01) were found in blastocysts derived from summer control oocytes.

**Figure 2:**
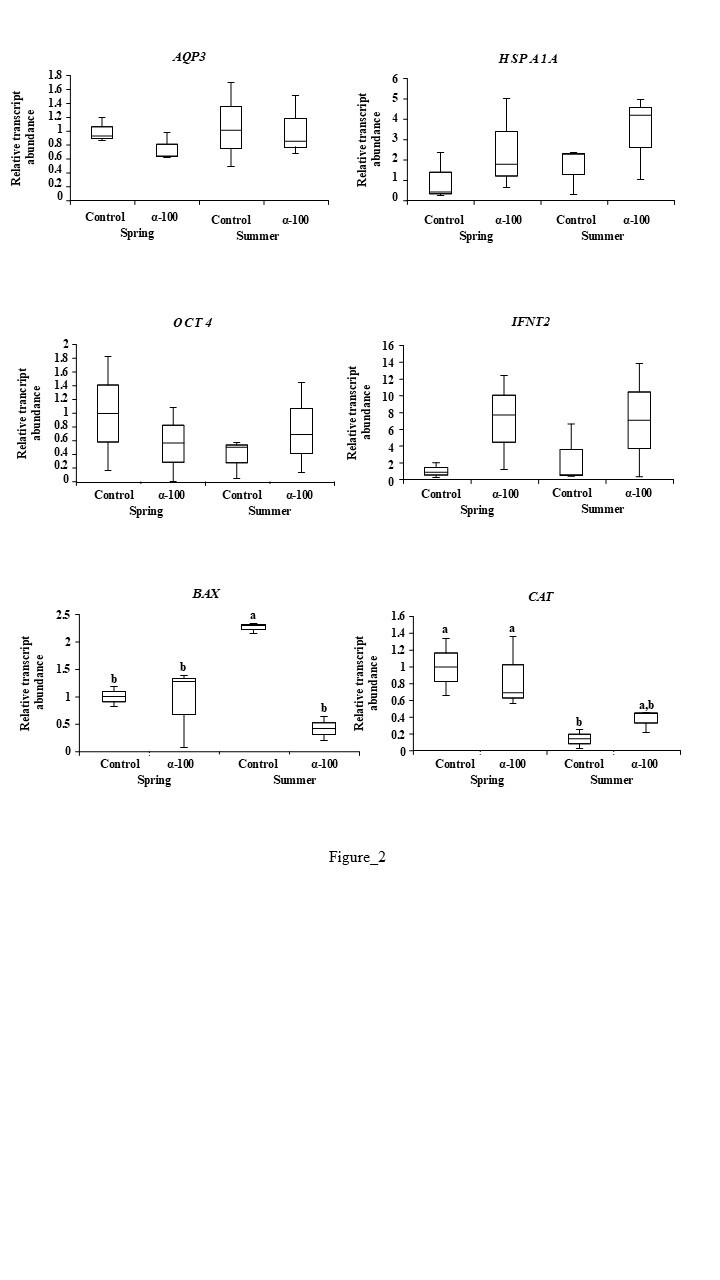
Effect of 100 µM of α-tocopherol supplementation during bovine oocyte *in vitro* maturation during spring and summer on the relative mRNA abundance for genes: *AQP3*, *HSPA1A*, *OCT4*, *IFNT2*, *BAX* and *CAT* for *in vitro* produced expanded blastocysts. The absence of letters in each bar represents no significant differences. Different letters (a, b) on each box indicate significant differences between treatments (*P < 0.05*).

Transcript abundance differences in expanded blastocysts were detected only for *BAX* and *CAT* genes (Figure 2). *BAX* transcript abundance was significantly higher in summer control blastocysts (*P*<0.05), with no differences among the other groups. Conversely, *CAT* transcript levels were the lowest in summer control blastocysts (*P<0.05)*.

## Discussion

The hypothesis that α-tocopherol reduces ROS production and apoptotic index in blastocysts produced in both seasons was partially confirmed. Our results demonstrate that α-tocopherol positively impacts the developmental potential of bovine oocytes collected during summer, exhibiting season-specific effects on oocyte maturation, fertilization, embryo yield, and quality. Conversely, spring-collected oocytes show no significant response to supplementation, suggesting that the specific benefits of α-tocopherol are limited to stressful seasonal conditions. In this study, the average THI during summer was around 73, indicating heat stress; while in spring the THI was 68: no heat stress risk (Schüller et al., 2014). Consistent with our findings, previous studies with oocytes collected from slaughterhouse (Yaacobi-Artzi et al., 2022) or *ovum pick-up* (Leite da Silva et al., 2020) during the summer, showed the lowest blastocyst rates. The environmental conditions prevailing during summer negatively affect embryo quality, with higher apoptotic indices and ROS levels compared to spring. Several studies reported increased apoptosis and ROS production in blastocysts derived from heat-shocked bovine COCs during IVM (Amaral et al., 2020; Balboula et al., 2013) or collected in summer (Ferreira et al., 2011; Rodríguez et al., 2022).

These results highlight the potential of α-tocopherol as a targeted intervention to improve oocyte and embryo quality under heat stress. Similarly, Maddahi et al. (2024) reported higher maturation and embryonic development rates in heat-stressed dairy cow oocytes supplemented with 100 µM α-tocopherol. Yashiro et al. (2015) reported that α-tocopherol treatment improved the proportion of blastocysts and increased the number of blastomeres in vitrified-warmed mature bovine oocytes. de Vasconcelos Franco et al. (2016) reported that α-tocopherol decrease lipid peroxidation in cryopreserved spermatozoa protecting sperm cells against oxidative damage during IVF. In addition, Natarajan et al. (2010) studied the supplementation of 200 μM α-tocopherol during *in vitro* embryo culture under low (5%) and high (20%) O_2_ tension. They found a significant improvement in the proportion and quality of blastocysts at 20% O_2_, where a higher production of ROS is expected (Guérin et al., 2001).

Our results suggest that α-tocopherol exerts its antioxidant effect under highly stressful conditions, as evidenced by reduced ROS production and decreased *BAX* gene expression in summer-collected oocytes. Notably, spring oocytes displayed low apoptosis index and *BAX* expression, even without supplementation, indicating their intrinsic competence and reduced need for antioxidant compensation. Taken together these results support the notion that α-tocopherol benefits gametes and preimplantation embryos under higher oxidative stress, as it reduces ROS and mitigates apoptosis-related gene expression. This is reinforced by recent research by Tripathi et al. (2023), which demonstrated that supplementation with 100 µM α-tocopherol during ovine oocyte IVM reduced oxidative stress by decreasing ROS levels and *BAX* expression, while increasing *SOD1* expression. Changes in transcript abundance for genes such as *SOD1* (Tripathi et al., 2023), *SOD2* (Báez et al., 2021) and *CAT* (Arias-Álvarez et al., 2018), caused by α-tocopherol, representing antioxidant responses, are related to decreased ROS levels. The *CAT* gene plays a crucial role by converting H_2_O_2_ into oxygen and water and mitigating apoptosis via *BAX* and *Caspase-3* downregulation (Khan et al., 2020). Together, these data underline the potential of α-tocopherol as a protective agent under stressful seasonal conditions.

## Conclusion

Our findings indicate that α-tocopherol supplementation during IVM significantly enhances blastocyst quality and yield of abattoir oocytes collected during summer, exerting its protective effect against oxidative damage by reducing ROS levels and apoptosis. Conversely, no significant effects were observed for spring oocytes, suggesting that the benefits of α-tocopherol supplementation during IVM are more evident for oocytes under stressful seasonal conditions, such as those experienced in summer.

## Data Availability

Research data is only available upon request.
